# Decrease of perforin positive CD3^+^γδ-T cells in patients with obstructive sleep disordered breathing

**DOI:** 10.1007/s11325-017-1602-6

**Published:** 2017-12-15

**Authors:** Richard Staats, Raquel Rodrigues, André Barros, Leonor Bacelar-Nicolau, Margarida Aguiar, Dina Fernandes, Susana Moreira, André Simões, Bruno Silva-Santos, João Valença Rodrigues, Cristina Barbara, António Bugalho de Almeida, Luis Ferreira Moita

**Affiliations:** 1 Departamento de Pneumologia, Hospital de Santa Maria, 1649-035 Lisbon, Portugal; 2 Instituto Gulbenkian de Ciência, Rua da Quinta Grande 6, 2780-156 Oeiras, Portugal; 3 Present Address: Departamento de Pediatria, Hospital de Santa Maria, Serviço de Genética Médica, 1649-035 Lisbon, Portugal; 4 Instituto de Medicina Preventiva and ISAMB, Faculdade de Medicina, Universidade de Lisboa, Avenida Professor Egas Moniz, 1649-028 Lisbon, Portugal; 5 Instituto de Medicina Molecular, Faculdade de Medicina, Universidade de Lisboa, Lisbon, Portugal

**Keywords:** Sleep-related breathing disorders, Obstructive sleep apnea, Perforin and granzyme-B-positive peripheral blood lymphocytes, Cancer, Obesity

## Abstract

**Introduction:**

Sleep related breathing disorders (SRBD) cause sleep fragmentation, intermittent hypoxia or a combination of both leading to homeostasis perturbations, including in the immune system. We investigated whether SRBD patients with or without intermittent hypoxia show substantial differences in perforin and granzyme-B positive peripheral blood lymphocytes.

**Methods:**

A total of 87 subjects were included and distributed as follows: 24 controls (C), 19 patients with respiratory effort related arousals due to increased upper airway resistance (UAR) without hypoxic events, 24 obese patients with obstructive sleep apnea (OSA) (oOSA), and 20 without obesity (noOSA). After polysomnographic recording, we analyzed in fasting blood samples routine hematologic and biochemical parameters and the percentage of lymphocytes containing the proteins perforin and granzyme-B (GrB). Kruskal-Wallis tests and a posteriori multiple comparisons were applied for statistical analysis of results.

**Results:**

Perforin-positive γδ-cells revealed significant differences between groups (*p* = 0.017), especially between the Control group and the oOSA (*p*-value = 0.04); the remaining SRBD groups also showed differences from the control (C vs UAR: *p* = 0.08; C vs noOSA = 0.09), but they did not raise to statistical significance. There were no differences among the SRBD groups. Granzyme-B cells were decreased in SRBD patients, but the differences were not statistically significant. No additional statistical significant result was found in the other investigated lymphocyte subsets.

**Conclusions:**

Obstructive sleep-disordered breathing is associated with a decrease in perforin-positive CD3^+^γδ-T cells. Although this finding was detected in lean patients without intermittent hypoxia, the reduction was only statistically significant in obese patients with severe OSA. Because CD3^+^γδ-T cells play an important role in the control of tumor cells, our findings are directly relevant for the study of the association of OSA and cancer.

**Electronic supplementary material:**

The online version of this article (10.1007/s11325-017-1602-6) contains supplementary material, which is available to authorized users.

## Introduction

Sleep is a critical adaptive behavior as it is essential for the maintenance of core homeostatic functions of an organism. A large body of evidence demonstrates the importance of sleep not only for various metabolic and inflammatory pathways, but also for humoral and cellular immune functions [1]. Sleep-related breathing disorders (SRBD) in general and the obstructive sleep apnea (OSA) in particular are extremely prevalent in the general population, and therefore constitute a public health problem. OSA is linked to increased mortality mainly due to higher prevalence of cardiovascular events [2]. The underlying mechanism is still under investigation but it is likely multifactorial. In addition to promoting a pro-atherogenic pattern in the peripheral blood [3], OSA increases nuclear factor kappa B (NFκB)-dependent endothelial inflammation [4]. Recent research has linked cytotoxic lymphocytes (CTL) to ateriosclerotic plaque instability and thus the risk of acute cardio-vascular events [5]. The main mechanism of cellular cytotoxicity is based on the secretion of the proteins perforin and granzymes into immunological synapse between a cytotoxic lymphocyte and a target cell [6]. Ultimately, the combination of perforin and GrB induces cell death by activating the caspase cascade leading to nuclear fragmentation and apoptosis [7]. While enhanced activity of cytotoxic lymphocytes constitutes an attractive idea to explain the increased cardiovascular risk in OSA, currently, there is little evidence to support a causal link between OSA in humans and an increased number or activity of perforin positive CD8^+^ lymphocytes [8]. Chronic inflammation is known to raise the risk of carcinogenesis [9]. Interestingly, OSA has also been linked to carcinogenesis, opening new possibilities to account for the increased mortality in OSA patients [10]. Intermittent hypoxia, sleep fragmentation, and increase in adipose tissue are three typical features of OSA patients. Any of them is known to influence inflammatory cascades and the immune system [11]. In this study, we tested the impact of each component of OSA in the immune response by analyzing perforin and GrB positive lymphocytes in non-obese patients with either intermittent hypoxia (OSA) or non-hypoxic sleep fragmentation.

## Material and methods

### Subjects

A total of 87 participants with an age between 20 and 59 years were included in this study. A total of 24 controls (C) were recruited from patients admitted to the sleep laboratory without detected sleep disorders, healthy members of the hospital employees or their relatives. All showed in the sleep study a respiratory disturbance index (RDI) and an oxygen desaturation index (ODI) < 5/h. Upper airway resistance (UAR) was defined as sleep fragmentation by mainly respiratory effort related arousals (RERAs). Patients of this group demonstrated a RDI > 5 and an ODI and apnea/hypopnea index (AHI) < 5/h. A total of 19 participants fulfilled the UAR criteria. Patients with obstructive sleep apneas (OSA) were defined by an apnea/hypopnea index (AHI) and ODI > 5/h. A total of 20 lean OSA patients with a body mass index (BMI) < 30 kg/m^2^ were detected (non-obese OSA or noOSA). As positive control group 24 obese OSA (oOSA) patients were included. Patients were not instructed to remain on a special diet before the sleep study. Eight oOSA patients were on regular cholesterol or diabetes mellitus therapy. Patients on immune system modulating therapies (e.g., recent vaccines or systemic corticoids) were excluded from this study. This project was approved by the Ethics Committee Review Board of Hospital de Santa Maria (Lisboa, Portugal), and all participants signed informed consent forms.

### Polysomnographic recordings

Sleep related events were investigated via standard polysomnography (PSG) Alice 5, Koninklijke Philips N.V. Philips Respironics, Murrysville, USA. The following parameters were recorded: F3; F4; C3; C4; O1; O2, M1, and M2. We used the standard referential montage of scalp electrodes against the contra-lateral mastoid electrode (e.g., C3/M2). Further parameters consisted of submental electrodes, strain gauges to record respiratory movements, EMG at both legs according to standard PSG procedures. Peripheral oxygen saturation was analyzed by pulse oximetry. The scoring of sleep and sleep-related events was based on the recommendation of the American Academy of Sleep Medicine published in 2007 [12].

#### Evaluation of sleepiness

Sleepiness was evaluated using the Stanford Sleepiness Scale (SSS) and the Epworth Sleepiness Scale (ESS). All patients completed the questionnaires in the morning following the polysomnographic recording.

#### Positive pressure therapy in OSA patients

All patients with a relevant OSA or UAR were invited to receive continuous positive airway (CPAP) therapy. A total of 42 patients (only with OSA) underwent another full polysomnographic recording with a CPAP titration protocol based on the existing recommendations [13].

### Blood analysis

Immediately after the diagnostic and therapeutic polysomnographic study fasting peripheral blood samples were obtained from a cubital vein and further analyzed in the laboratory.

#### Routine analysis

The following hematologic and biochemical parameters were investigated in all participants following the diagnostic PSG night: full peripheral blood cell count, hepatic enzymes (aspartate transaminase (AST), alanine transaminase (ALT) and gamma-glutamyltransferase (gGT)), renal parameters (creatinine, blood urea nitrogen (BUN)), standard metabolic parameters (uric acid, glucose, total cholesterol, low density lipoprotein (LDL), high density lipoprotein (HDL), triglycerides (TG), and C-reactive protein (CRP)).

#### Analysis of the cytotoxic proteins perforin (P) and granzyme-B (GrB)

##### Peripheral venous blood sample and Ficoll

Ethylenediaminetetraacetic acid (EDTA) anti-coagulated blood was collected directly after the end of the sleep study by peripheral venipuncture. Erythrocytes were lysed by adding 10 ml of FACS lysis buffer (BD Biosciences, Heidelberg, Germany). Peripheral blood mononuclear cells (PMNCs) were isolated by standard Ficoll gradient separation (FICOLL-PAQUE PLUS, GE Healthcare Biosciences).

##### Monoclonal antibodies

Anti-human CD3-phycoerythrine (PE) (clone UCHT1), CD4-PE (clone RPA-T4), CD8-PE (clone HIT8), CD16 indotricarbocyanine dye coupled to PE (PE-CY) (clone 4G8), CD56- PE-CY (cloneB159), perforin-fluorescein isothiocyanate (FITC) (clone γG9) and granzyme-B-FITC (clone GB11) were purchased from BD Biosciences, Heidelberg, Germany. Additionally, we used the CD3-PE-CY- (clone HIT3), and TCT-γδ-PE (clone B1) antibodies manufactured by BioLegend, San Diego, USA.

##### Cell surface and intracellular staining

**Fig. 1:**
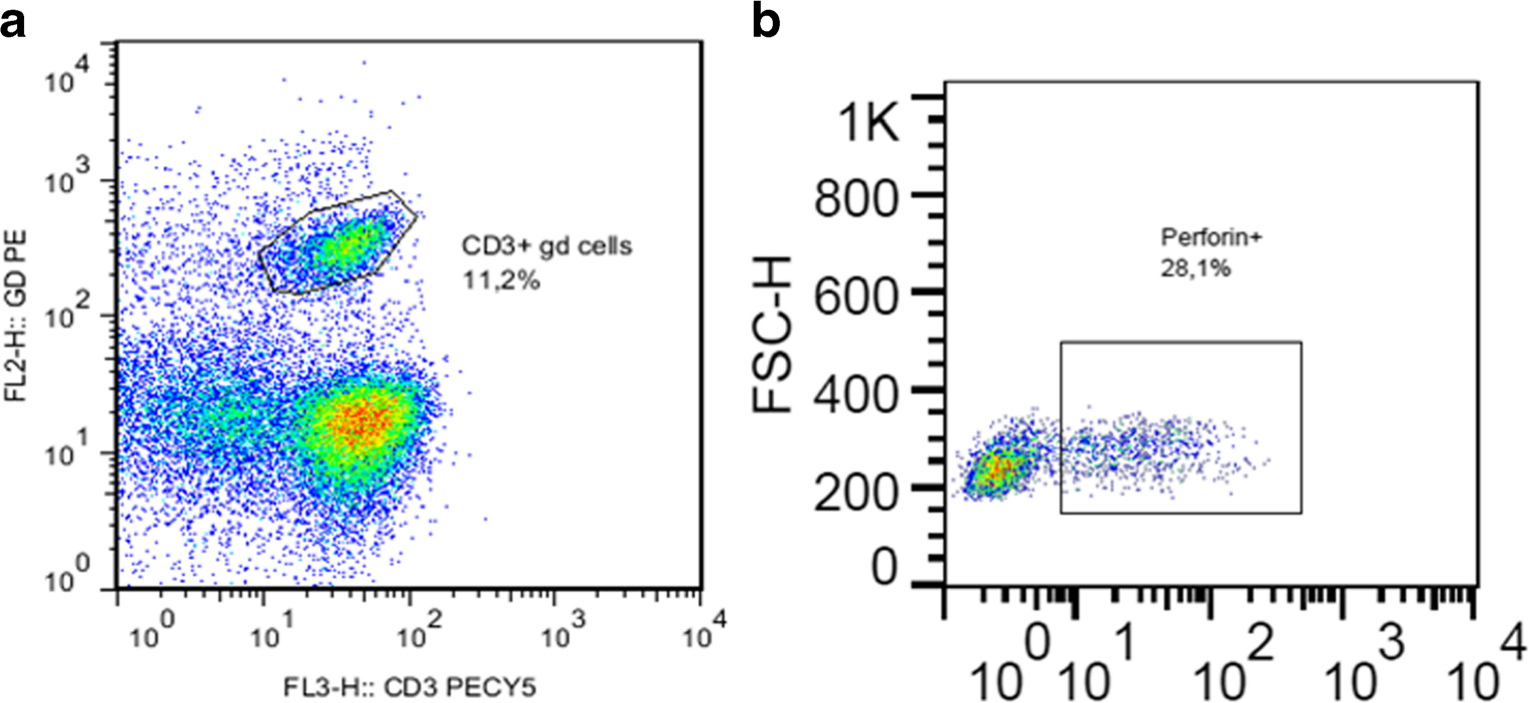
(A) Plot showing the gated lymphocytes in CD3-PECY5 on the x-axis versus γδ-PE on the y-axis. The graphic demonstrates an unusual high percentage of γδ T cells within the total lymphocytes. (B) Gating and calculating the percentage of the of perforin positive CD3^+^ γδ T lymphocytes

Peripheral mononuclear cells (pMNCs) were diluted to a concentration of 25 × 10^3^ cells/well and supplemented with phosphate-buffered saline (PBS) and 2% fetal calf serum (PBS/FCS buffer). After twice washing with PBS/FCS buffer and centrifuged at 2000 rpm, cells were incubated with 50 μg of a fluorochrome-labeled anti-human antibody solution followed by washing with the PBS/FCS solution. PMNCs were fixed with 2% paraformaldehyde in PBS for 30 min. After washing with PBS/FCS buffer cells centrifugation at 200 rpm, cells were permeabilized with 150 ml saponin 0.1% in PBS buffer for 10 min. Following washing and centrifugation with 2000 rpm / 3 min, antibodies against intracellular and cytotoxic proteins were added in a 25 μl/well saponin 0.1% solution. Cells were washed with the 0.1% saponin solution followed by two washed steps with PBS/FCS puffer. Membrane and intracellular antigen expression on pMNCs were in the following analyzed by flow cytometry (BD Biosciences, Heidelberg, Germany). Using double surface staining with PE or PE-Cy5labeled membrane antigens we were able to investigate the following lymphocyte subsets: CD3^+^CD4^+^, CD3^+^CD8^+^, CD3^−^CD8^+^, CD3^+^-γδ TCR^+^ (γδ-T cells), CD3^+^CD16^+^/CD56^+^ (natural killer T cells (NKT)) and CD3^−^CD16^+^/CD56^+^ (NK cells). FITC labeling permitted the additional analysis of the intracellular cytotoxic proteins perforin and granzyme-B (Fig. 1).

### Isolation of human peripheral blood γδ T cells

Blood from Buffy Coat units (50–70 mL) were obtained from healthy volunteers. Blood was centrifuged in Ficoll-Paque (Histopaque-1077; Sigma-Aldrich) for 35 min at 1.500 rpm and room temperature. The interphase containing peripheral blood mononuclear cells (PBMCs) was collected and washed in PBS. The desired TCR γδ^+^ T lymphocytes were labeled by incubation with hapten-conjugated anti-TCRγδ monoclonal antibody (Miltenyi Biotec GmbH), according to the manufacturer’s instructions. Further, cells were labeled with FITC-conjugated anti-hapten monoclonal antibody coupled to magnetic microbeads. The cell suspension was loaded onto a LS magnetic column (Miltenyi Biotec) and TCRγδ^+^ T lymphocytes were positively selected. γδ^+^ T lymphocytes were subsequently collected from the columns, following the manufacturer’s instructions, and resuspended in serum-free culture medium (OpTmizer-CTS) supplemented with 5% fetal bovine serum and 2 mM L-glutamine (Thermo Fisher Scientific) and 70 ng/mL of interleukin-2.

### Hypoxia induction in human peripheral blood γδ T cells

Isolated human γδ T cells were plated in 96-well plates and incubated at 37 °C, 5% CO_2_ and 19% O_2_ (normal condition), in Heracell™ 150i CO_2_ Incubator (Thermo Fischer), or incubated at 37 °C, 5% CO_2_ and 5% O_2_ (hypoxic condition), in New Brunswick Scientific - Galaxy 14S CO_2_ Incubator (Wolf Laboratories), for 24 h prior to cytokine production analysis and cell surface staining by FACS.

### Flow cytometry analysis

Cells were stained with the following antibodies from Biolegend: anti-human CD107a (H4A3), anti-human Granzyme B (GB11), and anti-human Perforin (#353312). Antibodies were coupled with APC and Pacific Blue fluorochromes. For intracellular cytokine production analysis, cells were either stimulated with PMA + Ionomycin + Brefeldin A for 4 h at 37 °C or incubated only with Brefeldin A and further stained with ebioscience IC kit according to the manufacturer’s instructions. Flow cytometry acquisition was performed on a LSR Fortessa (BD) and data was analyzed with FlowJo X software (Tree Star).

### Statistics

Descriptive statistics and hypothesis testing were performed using the Statistical Package for Social Science (SPSS) version 21 (SPSS Inc., Chicago, IL, USA) software, and multiple regression analysis was performed using R, version 3.4.1. (R Development Core Team, 2008).

Anthropometric data results are shown as mean (± SD). Sleep and laboratory results were not normal distributed and are shown as median (interquartile range). When two dependent samples were analyzed, the Wilcoxon signed rank test was applied. To compare between three or more different groups Kruskal-Wallis tests were applied and a posteriori Wilcoxon rank test for pairwise comparisons was performed, using a Holm-Bonferroni adjustment for the obtained *p-values*, due to the increasing probability of false positives inherent to multiple comparisons. A significance level of alpha 5% was used to determine statistical significance.

## Results

To investigate the effect of sleep disorders on cytotoxic immune system, we divided the study population into four groups, according to the diagnosis: in 24 of the total 87 included participants we found no sleep related breathing disorders (controls = C). In 19 patients, we detected sleep fragmentation due to increased upper airway resistance (UAR) but an apnea/hypopnea index within the normal range (< 5/H). A total of 20 non-obese and 25 obese patients (noOSA and oOSA, respectively) demonstrated an elevated obstructive sleep apnea/hypopnea (AHI) index (≥ 5/h). The latter three groups were considered to have sleep-related breathing disorders (SRBD).

### Anthropometric data

The anthropometric data is presented in Table 1. BMI was higher in the oOSA group (*p* < 0.001) when compared to the three other groups. Both OSA groups were older compared to controls (*p* = 0.006 for noOSA, *p* = 0.007 for oOSA. There were no evidences of any age differences between UAR group and the controls.

**Table 1:** Anthropometric data

	Controls (*n*:24)	UAR (*n*:19)	noOSA (*n*:20)	oOSA(*n*:24)
Age total [years]	37.71 ± 8.82 *#	41.42 ± 7.90	47.75 ± 8.42	47.04 ± 10.21
BMI [kg/m^2^] ± SD	25.33 ± 2.34#	25.44 ± 2.44§	25.87 ± 2.26°	33.23 ± 2.91

All results are presented by mean (± SD). Statistically significant results after adjustment, with a *p* < 0.05 are indicated as: Control vs. UAR: +, Control vs. noOSA: *, Control vs. oOSA: #, UAR vs. noOSA: ^. UAR vs. oOSA: §, noOSA vs. oOSA: °

### Sleepiness evaluation and polysomnographic results

The results of the sleepiness questionnaires and the polysomnographic recordings can be seen in Table 2. The results from the Epworth sleepiness scale (ESS) demonstrated OSA patients significantly sleepier when compared to controls (noOSA *p* = 0.008 and oOSA *p* = 0.004, respectively). Also, the slow wave sleep (N3) was significantly lower for the OSA groups compared to controls (noOSA: *p* = 0.008 e oOSA *p* = 0.019). Sleep fragmentation, defined by the arousal index (AI), was higher in all SRBD groups compared to controls (UAR *p* < 0.001, noOSA *p* < 0.001 and oOSA *p* < 0.001, respectively). Between the three SRBD groups, we detected differences between UAR and OSA groups (noOSA *p* = 0.002, oOSA *p* = 0.029).

**Table 2:** Polysomnography results and sleep questionnaires

	Controls	UAR	noOSA	oOSA
SE [%]	85.30 (79.98–88.70)	81.00 (74.95–88.20)	83.50 (75.65–86.97)	84.40 (78.90–89.20)
N3 [%]	19.65 *# (14.53–23.85)	14.80 (9.40–21.50)	10.60 (5.03–15.98)	6.90 (3.60–18.70)
R [%]	12.35 (9.30–15.38)	13.30 (10.35–16.05)	11.40 (8.45–15.55)	12.10 (7.30–14.30)
Arousal Index [/h]	18.70 + *# (14.10–21.00)	30.50^§ (26.50–38.60)	52.25 (41.50–59.77)	52.20 (32.70–72.40)
AHI [/h]	0.45 + *# (0.00–1.40)	2.10 ^§ (0.90–3.00)	24.50 ° (11.82–34.98)	51.00 30.90–76.60)
RDI [/h]	2.45 + *# (1.00–4.28)	10.70 ^§ (8.05–15.05)	34.20 (25.93–52.15)	63.40 (36.50–82.60)
Mean SpO2 [%]	96.00 *# (96.00–97.00)	96.00 ^§ (95.50–96.00)	94.50° (94.00–95.00)	93.00 (92.00–94.00)
ODI [/h]	0.70 + *# (0.30–1.48)	1.80 ^§ (1.35–3.50)	20.60 ° (12.53–41.65)	42.70 (28.40–69.30)
T 90 [%]	0.00 *# (0.00–0.00)	0.00 ^§ (0.00–0.00)	1.00 ° (0.30–4.00)	7.10 (2.20–18.10)
LMI [/h]	7.00 *# (4.65–11.38)	13.40 (7.90–20.70)	22.30 (11.53–34.35)	29.70 (15.00–46.00)
SSS [P]	2.00 (2.00–3.00)	2.00 (1.00–3.00)	3.00 (2.25–3.00)	3.00 (1.75–3.00)
ESS [P]	4.00 *# (2.00–6.50)	6.00 (2.00–10.00)	9.50 (6.50–14.75)	9.00 5.00–12.00)

All values are demonstrated as median (interquartile range). Statistically significant results after adjustment, with a *p* < 0.05 are indicated as: Control vs. UAR: +, Control vs. noOSA: *, Control vs. oOSA: #, UAR vs. noOSA: ^. UAR vs. oOSA: §, noOSA vs. oOSA: °

UAR: Upper Airway Resistance, noOSA: non-obese OSA, oOSA: obese OSA, SE: Sleep efficiency, N3: stage N3 sleep (slow wave sleep), AHI: apnea/hypopnea index, RDI: respiratory disturbance index, mean SpO2: mean peripheral saturation of oxygen. T90: percentage of oxygen saturation < 90%, LMI: Leg movement index of the lower limbs, SSS: Stanford Sleepiness Scale, ESS Epworth Sleepiness Scale

Hypoxia-related parameters including the apnea/hypopnea index (AHI), the oxygen desaturation index (ODI) and the percentage of peripheral oxygen saturation < 90% (T90) were significantly higher in both OSA groups when compared to either controls or UAR patients (Table 2). The respiratory disturbance index (RDI) was higher in all SRBD patients compared to controls (UAR *p* < 0.001, noOSA *p* < 0.001 and oOSA *p* < 0.001). Within the SRBD groups the RDI was found significantly higher in both OSA groups compared to UAR (noOSA *p* < 0.001 and controls *p* < 0.001). Following CPAP therapy, all polysomnographic parameters improved significantly (*p* < 0.05) with exception of sleep efficiency, that further decreased, R, and SSS. Results are demonstrated in Table 3 of the online supplementary material.

### Routine laboratory analysis

**Fig. 2:**
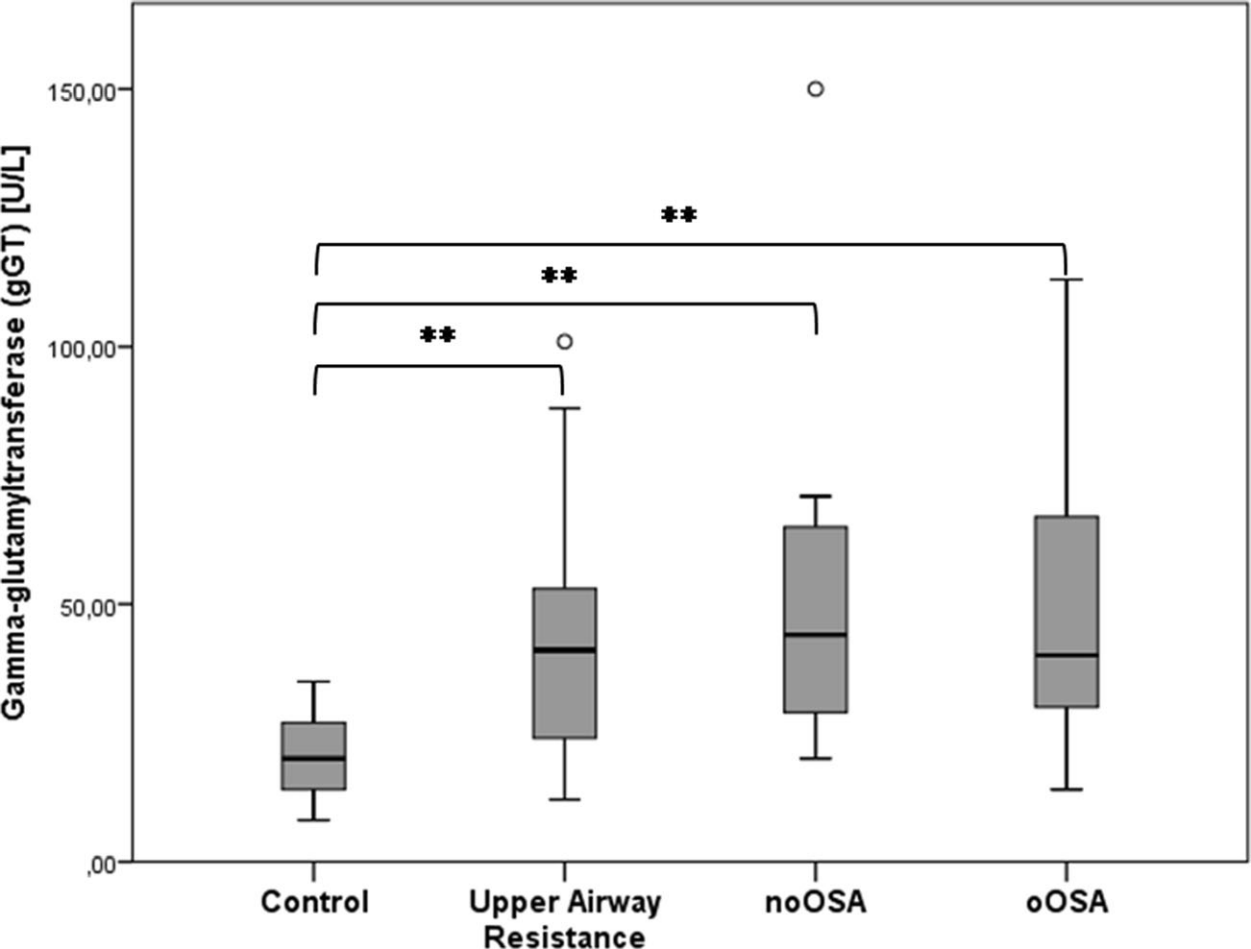
γGT levels in controls, upper airway resistance (UAR), lean obstructive sleep apnea (noOSA), and obese OSA (oOSA) patients. Statistical significance is indicated by *. Both OSA groups demonstrate a relatively similar pattern

Results for the routine blood analysis are listed in Tables 3 and 4. No significant difference was found in the full blood count analysis. The gGT level was twofold higher in all SRBD groups when compared to the control group (Fig. 2). Interestingly, median values were quite homogeneous between the three SRBD groups although the significance was higher in OSA patients (UAR *p* = 0.011; noOSA *p* = 0.001, oOSA, *p* < 0.001). ALT was also elevated in the SRBD patients, but only in oOSA patients statistically significant when compared to controls (*p* = 0.026) Serum glucose level was significantly higher in oOSA patients compared to controls (*p* = 0.013). We found no statistical significant differences in total cholesterol. The same applied for LDL. HDL was only significantly higher in the UAR group compared to oOSA (*p* = 0.004). Interestingly, the triglycerides were significantly higher in both OSA groups compared to either controls (noOSA *p* = 0.042; oOSA *p* = 0.015) or UAR patients (noOSA p = 0.013; oOSA p = 0.013). CRP level showed evidences of differences in the oOSA group when compared to controls (*p* = 0.021) and UAR patients (*p* = 0.021).

**Table 3:** Routine blood analysis

	Controls	UAR	noOSA	oOSA
Hb[g/dl]	15.70 (15.00–16.10)	15.10 (14.65–15.68)	15.10 (14.78–15.57)	15.00 (14.05–15.95)
HCT[%]	45.30 (41.60–47.40)	44.20 (42.77–45.27)	44.00 (42.20–46.35)	44.20 (41.30–46.95)
Leucocytes [10^9/L]	7.27 (5.75–8.27)	6.20 (5.48–6.90)	7.22 (5.76–9.88)	6.89 (5.85–8.19)
Lymphocytes [10^9/L]	2.44 (1.97–2.82)	2.25 (2.05–2.55)	2.53 (2.01–2.95)	2.79 (2.13–3.02)
Creatinine [mg/dl]	1.02 (0.89–1.12)	1.00 (0.95–1.14)	0.93 (0.88–1.00)	0.95 (0.88–1.06)
BUN [mg/dl]	18.46 (16.59–21.50)	18.69 (16.94–21.03)	17.76 (14.72–19.63)	18.46 (16.24–22.31)
AST [U/L]	23.00 (19.75–25.25)	23.00 (20.25–25.00)	25.00 (21.00–30.50)	27.50 (22.75–29.75)
ALT [U/L]	26.00 # (19.00–29.00)	29.50 (24.25–31.75)	36.00 (22.00–52.00)	38.00 (26.25–54.00)
gGT	20.00 + *# (14.00–27.00)	41.00 (24.75–52.00)	44.00 (29.00–65.00)	40.00 (30.75–64.75)
Glucose [mg/dl]	85.00 # (74.00–89.00)	82.50 § (75.50–86.00)	90.00 (78.50–111.00)	98.00 (89.00–104.00)
Colesterol tot [mg/dl]	177.00 (170.50–192.50)	212.50 (197.00–233.80)	218.00 (198.00–244.00)	197.50 (177.80–217.50)
LDL [mg/dl]	114.00 (105.50–121.50)	142.00 (113.50–153.80)	145.00 (115.00–167.00)	123.50 (105.00–143.00)
HDL [mg/dl]	42.00 (38.00–48.50)	47.50 § (44.00–58.00)	44.00 (37.00–50.00)	40.00 (36.25–44.75)
TG [mg/dl]	101.00 *# (68.50–120.50)	107.00 ^§ (75.25–124.75)	167.00 (152.00–252.00)	163.00 (125.00–221.20)
CRP [mg/dl]	0.04 # (0.04–0.13)	0.05 § (0.04–0.14)	0.12 (0.05–0.51)	0.30 (0.14–0.43)

All values are demonstrated as median (interquartile range). Statistically significant results after adjustment, with a *p* < 0.05 are indicated as: Control vs. UAR: +, Control vs. noOSA: *, Control vs. oOSA: #, UAR vs. noOSA: ^. UAR vs. oOSA: §, noOSA vs. oOSA: °

UAR: Upper Airway Resistance, noOSA: non-obese OSA, oOSA: obese OSA, Hb: hemoglobin, HCT: hematocrite, BUN: Blood Ureia Nitrogen, AST: Apartate Aminotransferase, ALT: Alanine Aminotransferase, gGT:gamma glutamyl transferase, LDL: Low-density lipoprotein Cholesterol, HDL: high-density lipoprotein cholesterol, TG: Triglycerides, CRP: C-reactive Protein

**Table 4:** Percentage of perforin positive lymphocytes within the lymphocyte subset

	Controls	UAR	OSAS non obese	OSAS obese
Total Perforin	23,60 (20.70–37.00)	19.40 (16.10–27.20)	22.60 (17.65–30.02)	24.90 (17.27–32.75)
CD3^+^P^+^/CD3^+^	12.40 (8.49–20.30)	13.75 (8.29–16.40)	14.65 (7.91–24.03)	15.70 (9.32–23.40)
CD3^+^CD4^+^P^+^/CD3^+^CD4^+^	1.60 (0.64–4.19)	2.28 (0.66–5.35)	1.63 (0.84–3.18)	1.60 (0.29–6.82)
CD3^+^CD8^+^P^+^/CD3^+^CD8^+^	28.50 (15.32–51.62)	28.20 (17.10–49.15)	24.80 (18.20–44.00)	30.55 (20.62–48.48)
CD3^−^CD8^+^P^+^/CD3^−^CD8^+^	66.35 (48.50–85.12)	85.80 (71.70–91.10)	79.75 (67.83–92.03)	84.90 (69.25–95.05)
CD3^+^γδP^+^/ CD3^+^gd	65.10 # (57.90–79.30)	46.20 (36.45–60.10)	45.95 (29.10–62.90)	38.75 (25.20–66.03)
CTLP^+^/CTL	90.90 (77.90–95.30)	86.15 (81.80–93.55)	86.70 (68.30–94.80)	86.45 (78.90–94.85)
NKP^+^/NK	98.20 (94.80–99.10)	96.00 (94.75–98.62)	95.20 (93.00–96.95)	95.75 (92.22–97.55)

All values are demonstrated as median (interquartile range). Statistically significant results after adjustment, with a *p* < 0.05 are indicated as: Control vs. UAR: +, Control vs. noOSA: *, Control vs. oOSA: #, UAR vs. noOSA: ^. UAR vs. oOSA: §, noOSA vs. oOSA: °

Gd cells: CD3^+^ ©™ T cells, CTL: CD3 + CD16 + CD56+ cell. NK: CD3-CD16 + CD56+ positive cells

### Lymphocyte subsets

The main results may be observed in table one of the supplementary information. Flow cytometric analysis revealed a lower percentage of NK cells in controls compared to noOSA and in UAR patients compared to the two OSA groups. However, such results were not statistically significant.

### Measurement of intracellular perforin and granzyme-B

**Fig. 3:**
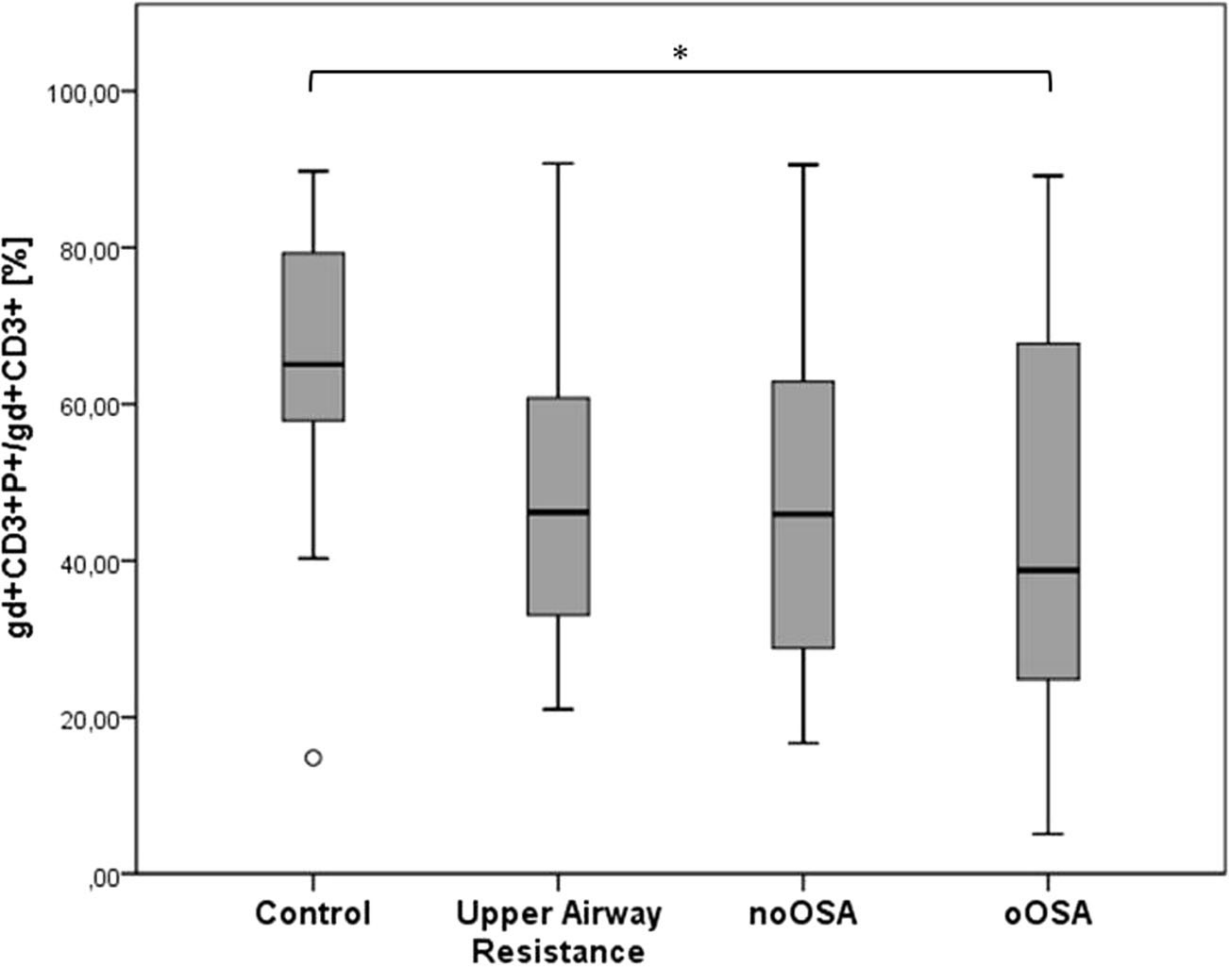
Perforin-positive CD3^+^γδ-T lymphocytes. Statistical significant results are indicated by *. All three sleep-disordered breathing groups demonstrate a similar distribution with a significant result in the statistical analysis for the oOSA when compared with the controls

To study the cytotoxic function, we focused on granzyme B-positive γδ-cells and perforin-positive γδ-cells (Fig. 1). While for granzyme-B cells there were not sufficient evidences among groups, perforin-positive γδ-cells revealed significant differences between groups (*p* = 0.01662, Fig. 3), especially between the control group and the oOSA (*p* = 0.04); the remaining SRBD groups showed differences from the control without reaching statistical significance (control vs UAR: *p* = 0.08; control vs noOSA = 0.09, Fig. 3).

These results are already an indication of an association between sleep-related breathing disorders and the cytotoxicity of the immune system; however, to fully test this association, we performed a correlation analysis between the perforin-positive γδ-cells and sleep-related parameters. Using the non-parametric Spearman Correlation Coefficient approach, we found correlations for AHI (Coef = −0.3568; *p* = 0.001), RDI (Coef = −0.3030; *p*= 0.007), Mean SpO2 (Coef = 0.4750; *p* < 0.001), T90 (Coef = −0.3880; *p* < 0.001), ODI (Coef = −0.3395; *p* = 0.002), SSS (Coef. = −0.2719; *p*= 0.022) and ESS (Coef. = −0.2550; *p* = 0.025).

These results suggest a relationship between sleep breathing-related disorders and the perforin-positive γδ-cells, but because there are other parameters that may influence the cytotoxicity of the immune system—age, BMI, metabolic function (and others)—we further analyzed the data to check whether the identified associations were maintained when such confounding variables are taken into consideration. To this end, we performed a multiple regression analysis, a method that is highly recommended for these situations. The multiple regression analysis results showed that even when several parameters were taken into consideration, SRBD-related variables such as Arousal Index (*p* = 0.049) and Mean SpO2 (*p* = 0.050) were still associated with the perforin-positive γδ-cells. The overall model also demonstrated to be better than a stochastic model (*p* = 0.039).

### Measurement of intracellular perforin and granzyme-B following therapy with continuous positive airway pressure (CPAP)

Following CPAP therapy the percentage of perforin positive cells within the lymphocyte subgroups increased with exception of the CD3^−^CD8^+^P^+^/CD3^−^CD8^+^ lymphocytes (Table 4 supplementary material). Only for the CD3^+^CD4^+^P^+^/CD3^+^CD4^+^ lymphocytes there was statistically relevant evidences of differences (*p* = 0.022). We found no statistically significant impact of the CPAP therapy in the GrB positive lymphocytes.

### In vitro measurement of degranulation capacity and intracellular perforin and granzyme-B following hypoxia of human γδ-T cells

**Fig. 4:**
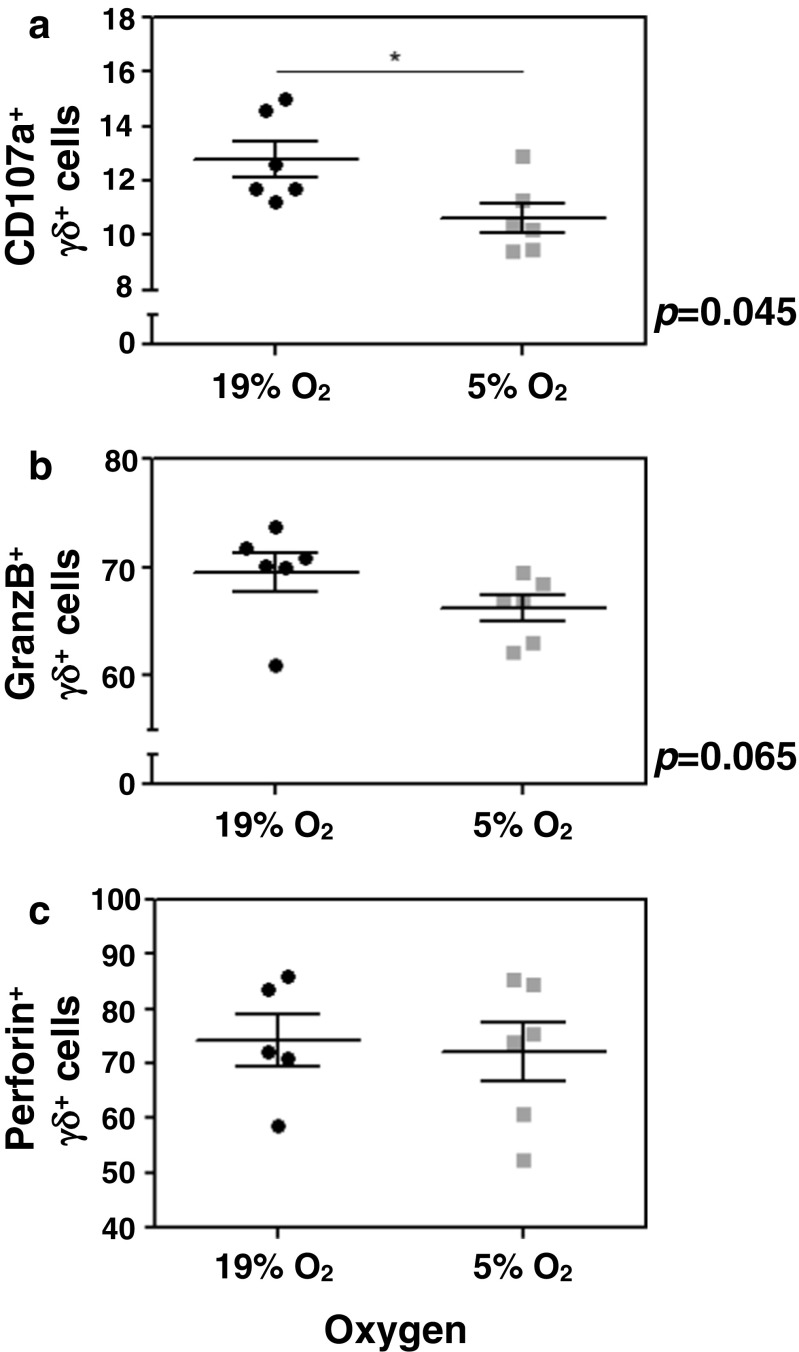
In vitro analysis of degranulation capacity (A), granzyme B, (B) and perforin (C) CD3^+^γδ-T lymphocytes, comparing normal levels of oxygen and hypoxia

To investigate whether intermittent hypoxia seen in sleep apnea patients is causally linked to decreased perforin levels in γδ-T cells, we isolated γδ-T cells from six independent anonymous donors from a blood bank and tested for their degranulation capacity, as measured by CD107a surface expression, perforin and granzyme B, comparing normal oxygen levels and hypoxic conditions. We observed a substantial and statistical significant decrease in the degranulation capacity of γδ-T cells in hypoxic conditions as evidenced by reduced levels of CD107a staining (Fig. 4a). We also found decreased levels of granzyme-B despite not being statistically significant (similarly to our patients, Fig. 4b), but no change in the perforin levels (Fig. 4c).

## Discussion

Sleep disturbances, including sleep-related breathing disorders (SRBD) in general and the obstructive sleep apnea (OSA) in particular, are known to contribute human homeostasis disruption [14] but the underlying mechanisms remain poorly understood. In OSA, the intermittent hypoxia due to either apneas or hypopneas and the sleep fragmentation due to the respiratory effort are the most important candidate mechanisms so far implicated.

In this study, we found that obstructive respiratory events including obstructive apnea, obstructive hypopnea, and respiratory effort-related arousals (RERA) are associated with a reduced percentage of perforin positive CD3^+^γδ T cells. Even in upper airway resistance patients (UAR) who by definition do not exhibit relevant oxygen desaturations, we saw some indications of this reduction, suggesting that sleep fragmentation, not only intermittent hypoxia, can influence the cytotoxic immune system.

Interestingly, therapy of OSA with continuous positive airway pressure (CPAP) increased the percentage of perforin-positive cells in most lymphocyte subpopulations (the differences were statistically significant only for CD3^+^CD4^+^ lymphocytes), suggesting that CPAP treatment is able to revert the observed decrease in perforin levels.

We have also investigated whether intermittent hypoxia could be causally linked to the changes observed and sufficient to explain the decrease in perforin levels found in the CD3^+^γδ T cells of patients. We did find that there are significant differences in the degranulation capacity of γδ T cells subjected to hypoxia as well as decreased levels of granzyme B, but no changes in the levels of perforin. These findings do suggest that hypoxia changes the degranulation capacity of CD3^+^γδ T cells in patients, but it is not sufficient to change the perforin level, which might be in direct connection to sleep fragmentation as suggested by the in vivo findings (UAR patients, who have no intermittent hypoxia, also have decreased perforin levels). The fact that we did not find changes in the levels of perforin might also reflect experimental limitations as it was not possible to mimic intermittent hypoxia and we were limited to continuous hypoxia. In addition, because we got samples from anonymous donors of a blood bank, we cannot exclude that some of the samples came from sleep apnea patients, as we know that the prevalence is very high in the general population [15]. By the current epidemiological data, it is possible that up to two out of the six samples came from donors with some level of SRBD. If this was the case, our in vitro experiment underestimated the real impact of hypoxia on the degranulation capacity of CD3^+^γδ T cells.

There have been few studies investigating the influence of OSA on the cellular immune system. Dyugovskaya and colleagues found an increased cytotoxic activity against endothelial cells for both γδ T cells and CD8^+^ lymphocyte in OSA patients [16, 17]. Although, at least at first glance, these results appear contradictory to ours, in fact they are not mutually exclusive. The described increased cytotoxicity of γδ T cells in OSA patients was TNF-α dependent and not based on the perforin/granzyme B pathway which is in general considered the faster and most effective mechanism of lymphocyte mediated cytotoxicity [6]. In our study, the percentage of perforin-positive CD8^+^ lymphocytes not expressing CD3 was higher in all three SRBD groups. This CD8^+^ lymphocyte subset is considered the most cytotoxic cells within all CD8 lymphocytes, especially when expressing CD56 [18]. Following one night of CPAP therapy, the percentage of perforin positive CD3^−^CD8^+^ lymphocytes decreased although the result was not statistically significant. Thus, in this small subset we observed the same trend as Dyugovskaya and colleagues.

It is currently unclear if the cytotoxic defense in OSA is increased [17], normal [19] or even decreased. Recently, Gaoatswe and colleagues demonstrated that circulating invariant natural killer T cells (iNKT) are reduced in OSA patients [20]. The more severe patients revealed the lowest number of iNKT lymphocytes. Incubation in a hypoxic environment increased apoptosis and decreased cytotoxicity of iNKT lymphocytes. Recent research provided evidence that OSA might increase the risk of cancer (reviewed by Gozal et al. [21]). Since invariant NKT cells are important for the anti-tumor response, Gaoatswe and colleagues suggested their results might contribute to explain the possible relationship between OSA and cancer. The γδ T cells are a critical component of the anti-tumor capabilities of the human immune response [22]. Therefore, our results constitute an important contribution to mechanistically explain the epidemiological relation between OSA and tumor diseases. The percentage of γδ T cells in the peripheral blood is small and usually less than 5%. However, γδ T cells demonstrate a high migratory capability with relevant accumulation in specific tissues [23, 24]. The decrease of perforin-positive γδ T cells in the peripheral blood that we describe might reflect a more important depression of the cytotoxic γδ T cells within epithelial tissues [25]. Interestingly, in a recent study Akparpour and colleagues showed that both intermittent hypoxia and sleep fragmentation reduces GrB^+^ CD8 lymphocytes within the tumor environment, in a mouse model of OSA [26].

Until now the epidemiological data for a possible OSA-cancer relation found mainly an association between nocturnal hypoxemia and cancer mortality [27]. The evidence regarding non-hypoxic sleep disturbances and tumor diseases is less established and mostly related to sleep restriction, insomnia, or shift work investigations with inconsistent results. Perhaps these conclusions can be explained by the lack of objective sleep data and abundance of subjective data based on questionnaires and sleep diaries. In the better-controlled animal studies both sleep fragmentation and intermittent hypoxia affected various components of the tumor progression including tumor growth or metastasis [28], which is in excellent agreement with the findings that we now report. The influence of obstructive sleep disordered breathing on tumor diseases was not the subject of the current study but our data suggests that both sleep fragmentation and hypoxia influence the cytotoxic immune defense independently and cumulatively.

Our study protocol did not include special diet recommendations prior to the sleep study, nor did we control for glucose or cholesterol affecting medication as we did not set out to investigate if RERAs are capable to influence the metabolic system. Therefore, the results regarding the glucose, cholesterol, and lipoprotein analysis should be interpreted cautiously. However, it appears noteworthy to mention that the gGT was significantly higher in all SRBD groups without any relevant difference between each other. To our knowledge, there is no evidence regarding the impact of respiratory effort related sleep fragmentation on the liver enzymes and this result deserves further investigation. CRP was higher in other oOSA groups when compared to either UAR patients or controls, confirming previous reports [29]. Also, it is of interest that controls and upper airway resistance patients had a significantly lower triglyceride level when compared to both OSA groups, although none of them was on regular medication. With the reservations mentioned, it is possible that apneas and hypopneas with relevant oxygen desaturation increase, independently from obesity, the pro-atherogenic role cholesterol and lipoprotein metabolism [30].

There are some limitations to the interpretation of the statistical results. The mean age was higher in the OSA groups when compared to controls. However, UAR patients had no significantly different age when compared to the other three investigated groups. In fact, these patients would be considered controls if less attention had been attributed to the RERAs classification. Any significant result in UAR patients compared to controls or OSA patients must be therefore considered significant. The immunosenescence described for the percentage of natural killer cells and the perforin related cytotoxicity might be of some concern [31]. Nevertheless, most evidence has been found in patients with an age above 60 years [32]. In our study, the inclusion was limited to an age below 60 years with a mean age of 47 years. It is thus questionable if imunosenescence already has any effect. Also of importance is the fact that there are only a small number of patients included in each group. However, with a total number of 87 included patients the study population is higher than most other studies investigating the relationship between SRBD and the immune system. In the future, to fully test the validity of our conclusions, a study on a more homogeneous group could be more effective.

In conclusion, this study analyzed the effect of hypoxic and non-hypoxic respiratory events on the perforin and granzyme-B positive lymphocytes. We found a decreased percentage of perforin positive CD3^+^γδ lymphocytes for the oOSA patients. Our results suggest that the sleep fragmentation and intermittent hypoxia observed in OSA cause changes in the cytotoxic potential of CD3^+^γδ lymphocytes which might be causally related to the increased cancer risk in these patients.

## Electronic supplementary material

ESM 1 (DOCX 17 kb)

## Abbreviations

AHI: Apneia/hypopneia index
ALT: Alanine transaminase
AST: Aspartate transaminase
CTL: Cytotoxic T lymphocytes (CD3 + CD16 + CD56+)
CRP: C-reactive protein
ESS: Epworth Sleepiness Scale
γδ T cells: Gamma-delta T lymphocytes
gGT: Gamma-glutamyltranspeptidase
GrB: Granzyme-B
HDL: High-density lipoprotein
LDL: Low-density lipoprotein
NK: Natural killer cells (CD3^−^CD16^+^CD56^+^)
NKT: Natural killer T cells (CD3^+^CD16^+^CD56^+^)
ODI: Oxygen desaturation index
P: Perforin
PBS: Phosphate-buffered saline
PSG: Polysomnography
RDI: Respiratory disturbance index
RERAs: Respiratory effort-related arousals
SRBD: Sleep-related breathing disorders
SSS: Stanford sleepiness scale
TG: Triglycerides

## References

[1] Siegel JM (2005). Clues to the functions of mammalian sleep. Nature.

[2] Marin JM, Carrizo SJ, Vicente E, Agusti AG (2005). Long-term cardiovascular outcomes in men with obstructive sleep apnoea-hypopnoea with or without treatment with continuous positive airway pressure: an observational study. Lancet.

[3] Kawano Y, Tamura A, Kadota J (2012). Association between the severity of obstructive sleep apnea and the ratio of low-density lipoprotein cholesterol to high-density lipoprotein cholesterol. Metabolism.

[4] Greenberg H, Ye X, Wilson D, Htoo AK, Hendersen T, Liu SF (2006). Chronic intermittent hypoxia activates nuclear factor-kappaB in cardiovascular tissues in vivo. Biochem Biophys Res Commun.

[5] Bobryshev, Y. V. (2005) Natural killer T cells in atherosclerosis, *Arterioscler Thromb Vasc Biol.* 25, e40; author reply e40 10.1161/01.ATV.0000161317.01678.75 15863714

[6] Krzewski K, Strominger JL (2008). The killer's kiss: the many functions of NK cell immunological synapses. Curr Opin Cell Biol.

[7] Trapani JA, Smyth MJ (2002). Functional significance of the perforin/granzyme cell death pathway. Nat Rev Immunol..

[8] Dyugovskaya L, Lavie P, Hirsh M, Lavie L (2005). Activated CD8+ T-lymphocytes in obstructive sleep apnoea. Eur Respir J.

[9] Federico A, Morgillo F, Tuccillo C, Ciardiello F, Loguercio C (2007). Chronic inflammation and oxidative stress in human carcinogenesis. Int J Cancer.

[10] Gozal, D., Farre, R. & Nieto, F. J. (2015) Putative links between sleep apnea and cancer: from hypotheses to evolving evidence. Chest 10.1378/chest.15-0634 PMC4631033 26020135

[11] Ryan S, Taylor CT, McNicholas WT (2005). Selective activation of inflammatory pathways by intermittent hypoxia in obstructive sleep apnea syndrome. Circulation.

[12] Iber C, A.-I. S., Chesson A, Quan SF (2007) The AASM manual for the scoring of sleep and associated events: rules, terminology and technical specifications, American Academy of Sleep Medicine, Westchester, Illinois

[13] Kushida CA, Littner MR, Hirshkowitz M, Morgenthaler TI, Alessi CA, Bailey D, Boehlecke B, Brown TM, Coleman J, Friedman L, Kapen S, Kapur VK, Kramer M, Lee-Chiong T, Owens J, Pancer JP, Swick TJ, Wise MS (2006). Practice parameters for the use of continuous and bilevel positive airway pressure devices to treat adult patients with sleep-related breathing disorders. Sleep.

[14] Krueger JM, Frank MG, Wisor JP, Roy S (2016). Sleep function: toward elucidating an enigma. Sleep Med Rev.

[15] Heinzer R, Vat S, Marques-Vidal P, Marti-Soler H, Andries D, Tobback N, Mooser V, Preisig M, Malhotra A, Waeber G, Vollenweider P, Tafti M, Haba-Rubio J (2015). Prevalence of sleep-disordered breathing in the general population: the HypnoLaus study. Lancet Respir Med.

[16] Dyugovskaya L, Lavie P, Lavie L (2003). Phenotypic and functional characterization of blood gammadelta T cells in sleep apnea. Am J Respir Crit Care Med.

[17] Dyugovskaya L, Lavie P, Lavie L (2005). Lymphocyte activation as a possible measure of atherosclerotic risk in patients with sleep apnea. Ann N Y Acad Sci.

[18] Addison EG, North J, Bakhsh I, Marden C, Haq S, Al-Sarraj S, Malayeri R, Wickremasinghe RG, Davies JK, Lowdell MW (2005). Ligation of CD8alpha on human natural killer cells prevents activation-induced apoptosis and enhances cytolytic activity. Immunology.

[19] Nakamura T, Chin K, Shimizu K, Kita H, Mishima M, Ohi M (2001). Acute effect of nasal continuous positive airway pressure therapy on the systemic immunity of patients with obstructive sleep apnea syndrome. Sleep.

[20] Gaoatswe G, Kent BD, Corrigan MA, Nolan G, Hogan AE, McNicholas WT, O'Shea D (2015). Invariant natural killer T cell deficiency and functional impairment in sleep apnea: links to cancer comorbidity. Sleep.

[21] Gozal D, Farre R, Nieto FJ (2016). Obstructive sleep apnea and cancer: epidemiologic links and theoretical biological constructs. Sleep Med Rev.

[22] Silva-Santos B, Serre K, Norell H (2015). gammadelta T cells in cancer. Nat Rev Immunol.

[23] Thielke KH, Hoffmann-Moujahid A, Weisser C, Waldkirch E, Pabst R, Holtmeier W, Rothkotter HJ (2003). Proliferating intestinal gamma/delta T cells recirculate rapidly and are a major source of the gamma/delta T cell pool in the peripheral blood. Eur J Immunol.

[24] Chennupati V, Worbs T, Liu X, Malinarich FH, Schmitz S, Haas JD, Malissen B, Forster R, Prinz I (2010). Intra- and intercompartmental movement of gammadelta T cells: intestinal intraepithelial and peripheral gammadelta T cells represent exclusive nonoverlapping populations with distinct migration characteristics. J Immunol.

[25] Vroom TM, Scholte G, Ossendorp F, Borst J (1991). Tissue distribution of human gamma delta T cells: no evidence for general epithelial tropism. J Clin Pathol.

[26] Akbarpour, M., Khalyfa, A., Qiao, Z., Gileles-Hillel, A., Almendros, I., Farre, R. & Gozal, D. (2016) Altered CD8+ T-cell lymphocyte function and TC1 cell Stemness contribute to enhanced malignant tumor properties in murine models of sleep apnea. Sleep 10.1093/sleep/zsw040 28364502

[27] Campos-Rodriguez F, Martinez-Garcia MA, Martinez M, Duran-Cantolla J, Pena Mde L, Masdeu MJ, Gonzalez M, Campo F, Gallego I, Marin JM, Barbe F, Montserrat JM, Farre R (2013). Association between obstructive sleep apnea and cancer incidence in a large multicenter Spanish cohort. Am J Respir Crit Care Med.

[28] Almendros I, Wang Y, Becker L, Lennon FE, Zheng J, Coats BR, Schoenfelt KS, Carreras A, Hakim F, Zhang SX, Farre R, Gozal D (2014). Intermittent hypoxia-induced changes in tumor-associated macrophages and tumor malignancy in a mouse model of sleep apnea. Am J Respir Crit Care Med.

[29] Nadeem R, Molnar J, Madbouly EM, Nida M, Aggarwal S, Sajid H, Naseem J, Loomba R (2013). Serum inflammatory markers in obstructive sleep apnea: a meta-analysis. J Clin Sleep Med.

[30] Lin, M. T., Lin, H. H., Lee, P. L., Weng, P. H., Lee, C. C., Lai, T. C., Liu, W. & Chen, C. L. (2014) Beneficial effect of continuous positive airway pressure on lipid profiles in obstructive sleep apnea: a meta-analysis. Sleep Breath 10.1007/s11325-014-1082-x PMC4559086 25450153

[31] Almeida-Oliveira A, Smith-Carvalho M, Porto LC, Cardoso-Oliveira J, Ribeiro Ados S, Falcao RR, Abdelhay E, Bouzas LF, Thuler LC, Ornellas MH, Diamond HR (2011). Age-related changes in natural killer cell receptors from childhood through old age. Hum Immunol.

[32] Le Garff-Tavernier M, Beziat V, Decocq J, Siguret V, Gandjbakhch F, Pautas E, Debre P, Merle-Beral H, Vieillard V (2010). Human NK cells display major phenotypic and functional changes over the life span. Aging Cell.

