# Supplementary material for: Decrease of perforin positive CD3+γδ-T cells in patients with obstructive sleep disordered breathing
Source: Sleep Breath. 2017 Dec 15;22(1):211–21. doi: 10.1007/s11325-017-1602-6 (PMC5835055; doi:10.1007/s11325-017-1602-6)
Supplement: Supplementary file 1 — (DOCX 17 kb) [file 11325_2017_1602_MOESM1_ESM.docx]

**Online supplement:**

Online supplementary table 1: Percentage of specific membrane antigen positive lymphocytes within total lymphocytes.

|  | C | UAR | noOSAS | oOSAS |
| --- | --- | --- | --- | --- |
| CD3^+^ [%]  of total lymphocytes | 68.90  (62.20 - 72.90) | 75.65  (67.3 - 80.85) | 70.55  (66.08 - 72.28) | 71.15  (65.03 - 74.58) |
| CD3^+^CD4^+^ [%]  of total lymphocytes | 42.80  (36.20-46.80) | 45.55  (40.73 - 52.15) | 42.70  (34.65 - 48.62) | 45.60  (39.70 - 49.45) |
| CD3^+^CD8^+^ [%]  of total lymphocytes^+^ | 21.80  (18.80-23.80) | 22.75  (20.90 - 25.27) | 20.90  (14.55 - 27.27) | 21.95  (17.25 - 26.20) |
| CD3^-^CD8^+^ [%]  of total lymphocytes ^+^ | 1.54  (0.75-1.88) | 1.32  (0.95-1.99) | 1.42  (0.93 - 2.25) | 2.30  (1.33 - 3.75) |
| CD3^+^gd [%]  of total lymphocytes | 2.85  (2.20-3.44) | 3.38  (2.02 - 4.28) | 2.94  (2.30 - 4.17) | 2.82  (2.40 - 3.49) |
| CTL [%]  of total lymphocytes | 1.88  (1.32-3.01) | 3.78  (1.78 - 5.30) | 3.78  (1.48 - 8.81) | 3.24  (2.03 - 4.71) |
| NK[%]  of total lymphocytes | 9.78  (6.24-12.20) | 6.85  (5.38 - 10.41) | 11.70  (10.18 - 13.39) | 11.35  (8.06 - 16.45) |

All values are demonstrated as median (interquartile range). Statistically significant results after adjustment, with a p<0.05 are indicated as: Control vs. UAR: +, Control vs. noOSA: *, Control vs. oOSA: #, UAR vs. noOSA: ^. UAR vs. oOSA: §, noOSA vs. oOSA: º.

CD3^+^gd T Cells: CD3^+^ γδ lymphocytes, CTL: CD3+CD16+CD56+ cell. NK: CD3-CD16+CD56+ positive cells.

Online supplementary table 2 Percentage of granzyme-B positive lymphocytes within the lymphocyte subset.

|  | C | UAR | OSAS non obese | OSAS obese |
| --- | --- | --- | --- | --- |
| Total Granzyme B | 20.40  (15.40-31.60) | 17.35  (11.68 - 24.60) | 21.60  (18.50-26.52) | 22.40  (15.80-33.02) |
| CD3^+^GrB^+^/CD3^+^ | 10.15  (8.32-20.68) | 9.67  (6.16-14.50) | 13.90  (7.97 - 22.03) | 12.35  (9.28 - 21.73) |
| CD3^+^CD4^+^GrB^+^/CD3^+^CD4^+^ | 1.58  (0.40 - 4.02) | 1.59  (0.41 - 3.37) | 1.64  (0.86 - 2.91) | 2.40  (0.36-5.33) |
| CD3^+^CD8^+^GrB^+^/CD3^+^CD8^+^ | 21.35  (12.55-42.27) | 23.95  (11.50 - 46.90) | 29.05  (22.52 - 36.30) | 30.95  (18.93-48.80) |
| CD3^-^CD8^+^GrB^+^/CD3^-^CD8 | 64.40  (33.17 - 82.30) | 73.10  (58.80 - 81.12) | 67.45  (57.60 - 84.75) | 74.60  (76.70 - 89.95) |
| CD3^+^gd GrB^+^/ CD3^+^gd | 50.30  (34.20-56.30) | 36.70  (30.00 - 44.17) | 49.55  (29.38 - 56.12) | 39.60  (25.95 - 55.08) |
| CTL GrB^+^/CTL | 82.00  (67.00 - 87.10) | 71.85  (58.08 - 87.95) | 83.95  (64.67 - 93.83) | 75.25  (61.38 - 89.47) |
| NK GrB^+^/NK | 82.60  (80.40 - 91.25) | 85.10  (80.08 - 90.30) | 90.00  (75.85 - 93.17) | 81.35  (70.40 - 92.72) |

All values are demonstrated as median (interquartile range). There was no statistically significant results after adjustment, with a p<0.05

Gd cells: CD3^+^ γδ T cells, CTL: CD3+CD16+CD56+ cell. NK: CD3-CD16+CD56+ positive cells

Online supplementary table 3: Results of OSA patient’s diagnostic polysomnography (PSG) and CPAP titration PSG.

|  | Diagnostic PSG | CPAP Titration PSG |
| --- | --- | --- |
| SE [%] | 82.55  (72.78 - 87.08) | 79.90  (67.10 - 88.08) |
| N3 [%] | 7.85  (1.10 - 17.33) | 18.20 *  (10.57-28.62) |
| R [%] | 11.65  (5.60-19.10) | 14.05  (10.20-17.95) |
| Arousal Index [/h] | 52.45  (40.92-59.08) | 25.45 *  (22.15 - 40.45) |
| AHI [/h] | 30.60  (20.12 - 43.92) | 4.70 *  (2.98 - 11.03) |
| RDI [/h] | 40.50  (28.52-54.23) | 10.25 *  (6.10-14.55) |
| Mean SpO2 [%] | 94.00  (93.00-95.00) | 96.00 *  (95.00-96.00) |
| ODI [/h] | 30.50  (18.10 - 45.55) | 4.50 *  (1.70-11.20) |
| T 90 [%] | 2.00  (0.43-6.63) | 0.10 *  (0.00-0.30) |
| LMI [/h] | 27.50  (15.35-35.10) | 12.20 *  (4.75-18.95 ) |
| SSS [P] | 3.00  (1.25-3.00) | 2.00  (1.00-3.00) |
| ESS [P] | 10.50  (8.00-14.00) | 8.50 *  (5.00-10.75) |

All values are demonstrated as median (interquartile range). Statistically significant values (p<0.05) are indicated by *.

SE: Sleep efficiency, N3: stage N3 sleep (slow wave sleep), AHI: apnea/hypopnea index, RDI: respiratory disturbance index, mean SpO2: mean peripheral saturation of oxygen. T90: percentage of oxygen saturation < 90%, LMI: Leg movement index of the lower limbs, SSS: Stanford Sleepiness Scale, ESS Epworth Sleepiness Scale. . With exception of sleep efficiency, R and SSS all analyzed parameters improved significantly following one night of CPAP therapy.

Online supplementary table 4: Percentage of perforin positive lymphocytes within the lymphocyte subset following diagnostic polysomnography and after CPAP titration.

|  | Diagnostic | CPAP Titration |
| --- | --- | --- |
| Total Perforin | 25.40  (17.38-36.27) | 27.95  (19.68-34.95) |
| CD3^+^P^+^/CD3^+^ | 16.00  8.50-25.23) | 16.80  (9.79-27.15) |
| CD3^+^CD4^+^P^+^/CD3^+^CD4^+^ | 1.78  (0.39-7.19) | 2.32 *  (1.12-7.31) |
| CD3^+^CD8^+^P^+^/CD3^+^CD8^+^ | 32.30  (22.73-54.00) | 76.80  (64.05-91.30) |
| CD3^-^CD8^+^P^+^/CD3^-^CD8^+^ | 79.35  (68.88-93.40) | 42.60  (25.93-56.75) |
| CD3^+^γδP^+^/ CD3^+^gd | 44.45  (27.77-71.78) | 41.75  (28.40-62.20) |
| CTLP^+^/CTL | 88.00  (78.90-95.53) | 92.15  (81.95-95.47) |
| NKP^+^/NK | 95.45  (92.30-97.28) | 96.45  (92.65-97.88) |

All values are demonstrated as medians (interquartile range). Statistically significant values (p<0.05) are indicated by *.

Gd cells: CD3^+^ γδ T cells, CTL: CD3+CD16+CD56+ cell. NK: CD3-CD16+CD56+ positive cells. Significant results were only seen for CD3^+^CD4^+^P^+^ lymphocytes with a small increase following CPAP therapy.

Online supplementary table 5: Percentage of granzyme B positive lymphocytes within the lymphocyte subset following diagnostic PSG and after CPAP titration PSG.

|  | Diagnostic | CPAP Titration |
| --- | --- | --- |
| Total Granzyme B | 22.90  (17.40-22.90) | 24.90  (17.60-32.20) |
| CD3^+^GrB^+^/CD3^+^ | 14.50  (9.87-23.60) | 15.50  (8.92-23.60) |
| CD3^+^CD4^+^GrB^+^/CD3^+^CD4^+^ | 2.24  (0.93-6.77) | 3.05  (0.69-6.61) |
| CD3^+^CD8^+^GrB^+^/CD3^+^CD8^+^ | 30.20  (21.00-46.60) | 35.40  (23.40-52.40) |
| CD3^-^CD8^+^GrB^+^/CD3^-^CD8 | 74.60  (59.70-84.80) | 72.00  (58.80-85.00) |
| CD3^+^gd GrB^+^/ CD3^+^gd | 40.00  (27.80-54.70) | 34.70  (24.40-55.20) |
| CTL GrB^+^/CTL | 85.10  (63.40-92.00) | 83.90  (61.20-93.40) |
| NK GrB^+^/NK | 83.10  (73.70-92.80) | 87.10  (74.40-91.20) |

All values are demonstrated as medians (interquartile range). Statistically significant values (p<0.05) are indicated by *.

Gd cells: CD3^+^ γδ T cells, CTL: CD3+CD16+CD56+ cell. NK: CD3-CD16+CD56+ positive cells.
